# Adoption of Technology in Older Adults in Mexico City: An Approach from the Technology Acceptance Model

**DOI:** 10.3390/brainsci15060632

**Published:** 2025-06-12

**Authors:** Itzel Julieta De la Peña-López, Elizabeth Acosta-Gonzaga

**Affiliations:** Unidad Profesional Interdisciplinaria de Ingeniería y Ciencias Sociales y Administrativas, Instituto Politécnico Nacional, Mexico City 08410, Mexico; idelapenal1000@alumno.ipn.mx

**Keywords:** technology acceptance model, older adults, digital inclusion, anxiety, self-efficacy, digital technology

## Abstract

Background/Objectives: Currently, older adults face significant digital exclusion due to a lack of technological skills, which limits their access to essential services and their social participation in an environment increasingly dependent on technology. This study aimed to analyze how technological anxiety and social influence affect the perceived usefulness, perceived ease of use, and adoption intention of technological tools among older adults in Mexico City using the Technology Acceptance Model (TAM). Methods: A survey was conducted with 70 older adults attending an event in Mexico City. Results: The findings confirm that, although perceived usefulness and ease of use remain pillars of technology use intention, technology anxiety acts as a critical barrier limiting adoption. At the same time, social influence has a dual effect: on the one hand, it facilitates the perception of ease of use; on the other, it diminishes the perception of usefulness when support becomes pressuring or impatient. Conclusions: These results underscore the need to design interventions that reduce anxiety, strengthen digital literacy, and promote empathetic and motivating social support, thereby effectively enhancing technology adoption among older adults.

## 1. Introduction

In recent years, the use of technology has increased exponentially. However, there are differences in the digital knowledge and skills necessary to interact effectively with technology among the entire population, which reveals the existence of a digital divide. This gap is defined as inequality in access, use and exploitation of information and communication technologies (ICTs) between different sectors of the population [1]. In Mexico, the digital divide is shaped by socioeconomic factors such as age, education level, income, occupation, digital literacy, and geographic location [2].

Older adults, in particular, are facing significant challenges in integrating into the digital world. Many of them lack the skills and knowledge necessary to use digital technologies, which puts them at a disadvantage [3]. In 2023, 42.9% of people over 54 years of age in Mexico were not connected to the digital world, with the lack of digital skills being one of the main barriers [4]. This exclusion limits their participation in society and restricts access to essential services, further affecting businesses by reducing the potential benefits of ICT adoption [5].

The consequences of digital exclusion extend beyond social participation, impacting critical areas such as healthcare, employment, and overall well-being in an increasingly technology-dependent society [6,7]. Despite extensive international research on the digital divide among older adults, there remains a notable gap in studies focusing on the Mexican context. Furthermore, limited research has explored the specific factors influencing older adults’ intention to adopt digital technologies in Mexico.

To address this issue, this study applied the Technology Acceptance Model (TAM), originally proposed by Davis [8], as a theoretical framework. TAM explains technology adoption through two primary constructs: perceived usefulness, which refers to the extent to which an individual believes that using technology will enhance their performance, and perceived ease of use, which relates to the perception that technology is simple to learn and operate.

Additionally, TAM allows for the inclusion of external variables that can influence technology adoption, such as social influence and technological anxiety. Social influence encompasses the impact of close relationships, family, friends, or caregivers on an individual’s decision to use technology. In contrast, technological anxiety refers to the fear or discomfort associated with using digital devices, which can act as a significant barrier to adoption. Prior research has confirmed that these factors play a crucial role in shaping technology adoption among older adults, demonstrating that perceived usefulness and ease of use remain central to their willingness to engage with digital tools [9,10,11].

Based on this, the following research question is posed: How do technological anxiety and social influence affect older adults’ willingness to use technology in Mexico City?

The main objective of this study was to identify how factors such as technological anxiety and social influence impact perceived usefulness, perceived ease of use, and the behavioral intention of older adults in Mexico City to adopt digital technologies.

Based on the findings, recommendations have been proposed to promote the digital inclusion of this population, helping to reduce the technological gap by adapting technologies to their specific needs.

## 2. Technology Acceptance Model (TAM)

Over time, several models and theories have been developed in order to explain the acceptance and adoption of technology, one of the most prominent being the Technology Acceptance Model (TAM). Proposed by Davis [8], this model is based on earlier theories such as Ajzen and Fishbein’s [12] Theory of Reasoned Action (TRA) and Ajzen’s [13] Theory of Planned Behavior (TPB). Both TRA and TPB posit that human behavior is guided by rational decision-making, where an individual’s intention to engage in a specific action is influenced by their attitudes and perceived control over the behavior [9].

The TAM states that technology acceptance is primarily driven by two factors: perceived usefulness (PU) and perceived ease of use (PEU). Perceived usefulness refers to the degree to which a person believes that using a particular technology will improve his or her performance on a specific task, while perceived ease of use refers to the perception that using the technology will be effortless. According to the model, the greater the perception of usefulness and ease of use, the greater the probability of technology adoption. Specifically, if a person perceives a technology as useful and easy to use, they are more likely to develop a positive attitude toward it, thus increasing their intention to use it [10].

We selected the original TAM because its framework is both refined and resilient, enabling us to focus on technology anxiety and social influence, constructs extensively validated within this model [14]. Relying on this well-established approach allows for a targeted analysis consistent with our exploratory design and sample size.

## 3. Related Works

The TAM has emerged as a foundational framework for examining the adoption of digital technologies, particularly among older adults who frequently encounter structural and cognitive barriers to digital inclusion. Owing to its validity and adaptability, TAM has been widely employed to assess key determinants of technology use in aging populations.

Empirical applications of TAM confirm its cross-cultural relevance. In Spain, Martín García et al. [9] demonstrated that perceived usefulness and ease of use significantly predicted older adults’ intention to adopt digital technologies. Guner and Acarturk [10], extending TAM in Türkiye, incorporated user interface design, social influence, and personal satisfaction, emphasizing the interplay of psychological and environmental variables. Similarly, Yau and Hsiao [11] validated TAM in Taiwan, highlighting that digital engagement not only supports adoption but also enhances emotional well-being and social connectedness.

TAM has also been applied to healthcare technologies. Van Houwelingen et al. [15] found that older adults’ acceptance of video consultations in the Netherlands was shaped by effort expectancy, privacy concerns, and self-efficacy. Their study further emphasized the role of digital literacy, prior experience, and family support as enabling factors. In response to the unique characteristics of aging users, models such as the Senior Technology Acceptance Model (STAM) have integrated health status, cognitive abilities, and socioeconomic context [16]. Alexandru et al. [17] used STAM to explain mobile phone use in later life, stressing the importance of ease of learning, social support, and personal context.

Recent studies have continued to underscore technology anxiety and social influence as pivotal determinants of digital adoption among older adults. Drawing on the TAM and its variants, researchers have confirmed that anxiety, often rooted in limited prior experience and lower educational levels, significantly hinders both perceived ease of use and perceived usefulness [18,19]. For instance, Sun et al. [20] demonstrated that targeted interventions reducing anxiety through hands-on training and intuitive interfaces mediated the link between perceived usefulness and willingness to adopt smart health devices. Similarly, Felber et al. [21] found that age-friendly design features, such as simplified navigation and clear feedback, significantly alleviated users’ apprehensions, thereby boosting acceptance rates.

Equally critical is the role of social influence, which operates through family members, peers, and professional caregivers to shape older adults’ technology attitudes. Lu and Tsai-Lin [22] reported that encouragement from adult children and community volunteers directly enhanced perceived usefulness, while Jang et al. [23] observed that positive testimonials from peers increased perceived ease of use. Toros et al. [24] further noted that gerontechnology self-efficacy, strengthened by supportive social networks, moderated the relationship between perceived usefulness and adoption intentions, highlighting the synergistic effects of social support and personal confidence.

Despite these global insights, technology acceptance among older adults in Mexico remains under-researched. Although ICTs hold transformative potential for this demographic, barriers such as low digital skills and pervasive distrust persist [25]. Gutiérrez et al. [26] evaluated an interactive television (iTV) platform designed for older adults, finding high perceived utility but persistent difficulties in system navigation, underscoring the need for context-specific interventions.

Collectively, these findings validate the continued relevance of TAM and its extensions in aging research. However, further investigation into contextual variables, such as cultural attitudes, policy environments, and socioeconomic disparities, is essential to designing inclusive digital solutions that address the diverse needs of older populations.

## 4. Theoretical Framework

The TAM has been widely validated within diverse contexts and populations, making it a broad theoretical framework for analyzing technology acceptance. Next, each of the constructs of this model is described, as well as the hypotheses formulated from the review of previous studies.

### 4.1. External Variables

External variables indirectly influence both the intention to use and the actual use of technology by shaping perceptions of usefulness and ease of use. These variables include individual user characteristics, system features, and contextual or organizational factors [8]. In the case of older adults, two aspects are particularly relevant: technology anxiety and social influence.

Technology anxiety can create significant barriers to adoption by making technology appear difficult or inaccessible [14]. At the same time, social influence understood as the perceived expectations of people close to the individual, also plays a critical role, especially when family and friends either encourage or discourage the adoption of digital tools. Understanding these variables is essential for designing interventions that not only improve user experience but also reshape societal perceptions around aging and digital competence.

#### 4.1.1. Technology Anxiety

Technology anxiety refers to the fear or discomfort some individuals feel when interacting with digital technologies. Within the Technology Acceptance Model (TAM), this type of anxiety has been identified as a factor that reduces perceived ease of use and usefulness, thus hindering adoption [14].

Among older adults, this anxiety is often heightened by unfamiliarity with digital environments or negative past experiences. Several studies have confirmed its impact: Jøranson et al. [27] found that anxiety lowers the willingness to adopt new technologies; Froment and García González [25] observed that higher anxiety is associated with greater perceived difficulty; and Tsai et al. [28], in the context of health technologies, found that anxiety negatively affects both perceived ease of use and usefulness.

Based on these findings, the following hypotheses are proposed:

**H1.**  *Technology anxiety has a negative effect on perceived usefulness among older adults.*

**H2.**  *Technology anxiety has a negative effect on perceived ease of use among older adults.*

#### 4.1.2. Social Influence

Social influence refers to the extent to which individuals perceive that important people in their lives, such as family, friends, or colleagues, believe that they should use a particular technology. According to Venkatesh et al. [14], this factor significantly shapes technology adoption, as users are often influenced by the opinions and expectations of those around them.

For older adults, support or resistance from their social network can strongly impact their willingness to engage with technology. When family and peers encourage technology use, older adults are more likely to perceive it as useful and easy to use, increasing the likelihood of adoption.

Olschewski et al. [29] found that social influence and technology readiness play a critical role in technology adoption within the TAM framework. Choudrie et al. [30] reported that older adults often rely on the guidance of close family members when adopting new technologies. Similarly, Hoque and Sorwar [31] identified social influence as a key driver of mobile health technology adoption, while Guner and Acarturk [10] showed that it positively impacts perceived ease of use.

Based on these insights, the following hypotheses are proposed:

**H3.**  *Social influence has a positive effect on perceived usefulness among older adults.*

**H4.**  *Social influence has a positive effect on perceived ease of use among older adults.*

### 4.2. Perceived Usefulness

Perceived usefulness, as defined by Davis [8], refers to the degree to which a person believes that using a particular technology would enhance their performance in a specific task. Within the TAM, this construct plays a central role in determining an individual’s intention to use technology. For older adults, perceived usefulness is especially important, as they are more likely to adopt technologies they consider beneficial or relevant to their daily lives.

Shin et al. [32] explored the use of assistive technologies for daily living and found that perceived usefulness significantly influenced the behavioral intention of older adults to adopt such tools. Their study emphasized that when older users recognize the clear benefits of the technology, they are more inclined to integrate it into their routines. Similarly, Ma et al. [33] highlighted perceived usefulness as a key factor in the acceptance of new technologies among older adults. Their findings indicate that older individuals are more receptive to technologies they find useful, particularly those they are already familiar with, which lowers resistance and enhances acceptance.

In this context, perceived usefulness not only facilitates the initial interest in using technology but also reinforces long-term engagement by aligning technological functions with users’ expectations and needs.

Based on these findings, the following hypothesis is proposed:

**H5.**  *Perceived usefulness has a positive effect on the intention of older adults to use technology.*

### 4.3. Perceived Ease of Use

Perceived ease of use, as defined by Davis [8], refers to the extent to which an individual believes that using a technology will require minimal effort. Within the TAM, this variable plays a central role, especially for older adults, who often face greater challenges with digital tools due to limited familiarity or confidence.

Several studies confirm the relevance of this factor. Zin et al. [34] found that ease of use significantly improves older adults’ attitudes toward digital health devices. Yau and Hsiao [11] demonstrated that systems perceived as easy to use are also viewed as more useful, reinforcing the connection between usability and perceived value. Aguilar-Flores et al. [35] similarly concluded that perceived ease of use positively influences the intention to adopt ICT among older adults in Chile.

These findings suggest that when technologies are intuitive and user-friendly, older adults are more likely to accept and integrate them into their daily lives.

Based on this evidence, the following hypotheses are proposed:

**H6.**  *Perceived ease of use has a positive impact on perceived usefulness for older adults.*

**H7.**  *Perceived ease of use has a positive impact on older adults’ intention to use technology.*

### 4.4. Behavioral Intention to Use

Behavioral intention to use refers to the individual’s motivation or plan to adopt a technology in the near future. According to Davis [8], this intention is primarily shaped by perceived usefulness and perceived ease of use, and it strongly predicts actual usage behavior.

Tsai et al. [28] found that both perceived usefulness and ease of use significantly influenced older adults’ intention to adopt a smart clothing system designed for heart monitoring. Participants were more inclined to adopt the technology when they saw it as helpful and easy to operate. Similarly, Dogruel et al. [36] emphasized that perceived usefulness plays a central role in the adoption of entertainment technologies among older adults, especially when these tools are also perceived as easy to use and enjoyable. Their findings confirm that ease of use enhances perceived usefulness, which in turn strengthens the behavioral intention to use the technology.

Furthermore, perceived ease of use also plays an important role, as it is a predictor of perceived usefulness. That is, older adults only enjoy technology if they perceive it as easy to use. Therefore, ease of use is a key factor in fostering the intention to use interactive technologies.

### 4.5. Research Hypotheses

Based on the theoretical framework presented in Section 4.1, Section 4.2, Section 4.3 and Section 4.4, the followingresearch hypotheses are proposed in Table 1:

**Table 1:** Research hypotheses.

	Hypothesis
H1	Technology anxiety has a negative effect on perceived usefulness among older adults.
H2	Technology anxiety has a negative effect on perceived ease of use among older adults.
H3	Social influence has a positive effect on perceived usefulness among older adults.
H4	Social influence has a positive effect on perceived ease of use among older adults.
H5	Perceived usefulness has a positive effect on the intention of older adults to use technology.
H6	Perceived ease of use has a positive impact on perceived usefulness for older adults.
H7	Perceived ease of use has a positive impact on older adults’ intention to use technology.

**Figure 1:**
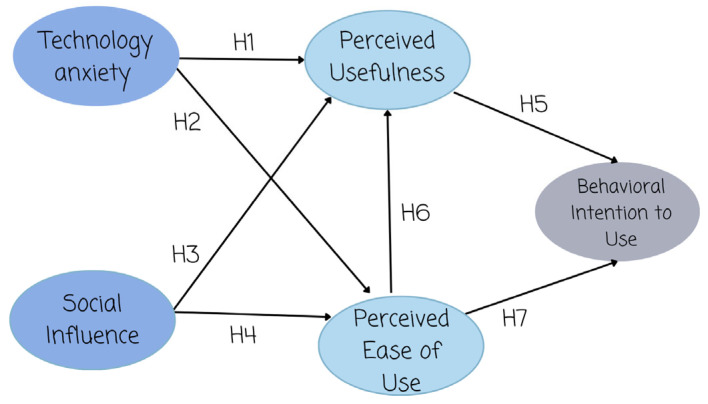
Conceptual model.

These hypotheses serve as the foundation for the conceptual model illustrated in Figure 1 and guide the empirical analysis of technology acceptance among older adults.

## 5. Materials and Methods

For this study, a quantitative research approach was conducted, which is characterized by the collection of data to test pre-established hypotheses, using numerical measurements and statistical analysis to identify behavior patterns and validate theories [37].

### 5.1. Data Collection

This study was conducted with a sample of 70 participants. Although this is a moderate sample size, it meets the methodological requirements for Partial Least Squares Structural Equation Modeling (PLS-SEM), which is suitable for small to medium samples. According to Wang and Wang [38], a minimum of five cases per item is recommended to ensure stable and reliable parameter estimates. In this regard, the sample was sufficient to achieve a robust model fit and support the validity of the results obtained.

The present study employed a non-probabilistic convenience sampling method, which is commonly used in exploratory and applied research when access to the entire population is limited or impractical [37,39]. This approach allowed to select participants based on their availability and willingness to participate. Although this sampling method limits the generalizability of the findings, it is considered appropriate for initial investigations aimed at identifying patterns and testing theoretical models such as the TAM in specific demographic groups [40].

### 5.2. Instruments

In order to measure the level of technology acceptance among older adults, a survey based on the TAM was applied, consisting of 14 items identified through a literature review. The survey was conducted during the Senior Citizens Festival, held on 5–7 April 2024, at the World Trade Center, Mexico City.

To ensure consistency in participants’ understanding, they were asked questions about the digital tools we referred to in order to assess their relationship with technology. These tools included the use of email, online banking, social networks, WhatsApp, Microsoft Word, Excel, PowerPoint, and videoconferencing platforms such as Zoom, Google Meet, and Microsoft Teams either on their computer or smartphone.

In this research, fundamental ethical principles were considered to ensure respect and protection of participants’ rights, such as informed consent, confidentiality, and data protection, following the recommendations of Creswell and Creswell [41] and Flick [42]. Participants were informed about the study’s objectives and procedures, emphasizing the voluntary nature of their participation, the option to withdraw at any time, and the protection of their personal data.

Each item of the survey was evaluated on a five-point Likert scale (Table 2), where the response options ranged from strongly disagree (1) to strongly agree (5).

### 5.3. Data Analyses

To test the proposed conceptual model, the Structural Equation Modeling (SEM) statistical technique was applied using SMART PLS software, version 4.0. This analysis aimed to assess the validity and reliability of the observed variables and constructs. Reliability was verified using Cronbach’s alpha coefficient (α), with a minimum acceptable value of 0.70 [45].

Convergent validity is observed when the indicators of a given construct exhibit a high correlation among themselves. A widely used method to evaluate this validity is the average variance extracted (AVE) [46]. The AVE reflects the proportion of variance that the indicators share with the latent construct. According to Chin and Marcoulides [47], an AVE value greater than 0.5 indicates that the construct explains more than half of the variance of its indicators, which is considered acceptable.

Composite reliability (CR) helps assess the effectiveness of a set of indicators in jointly measuring the same concept or theoretical construct. Unlike Cronbach’s alpha, this measure focuses more on the specific contribution of each indicator, making it more precise and flexible. A CR value above 0.7 typically indicates that the indicators function consistently [48,49].

To evaluate the quality of model fit in the structural model, the explained variance (R^2^) was used, an essential metric in structural analysis that indicates the percentage of variance in a dependent variable that can be explained by the independent variables included in the model. In other words, R^2^ reflects how well the model captures the theoretical relationships among the studied constructs. According to Hair et al. [48], an R^2^ value above 0.10 is acceptable for exploratory research, while values exceeding 0.50 are characteristic of models with high explanatory power in confirmatory studies.

## 6. Results

### 6.1. Descriptive Results

The analysis began with a presentation of the descriptive statistics of the sample. A total of 70 older adults participated in the study. The mean age of the participants was 69.36 years. In terms of gender, 58.6% were female (*n* = 41) and 41.4% were male (*n* = 29). This distribution reflects the broader demographic trend in Mexico, where women represent a higher proportion of the older population.

**Figure 2:**
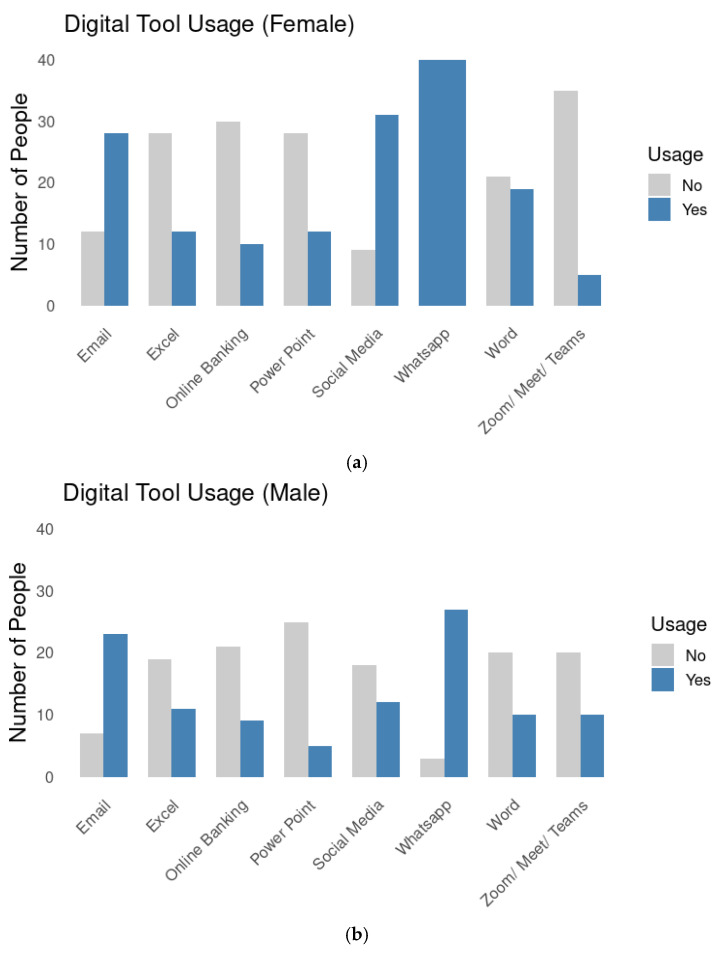
(**a**). Bar chart illustrating the frequency of digital tool use among older women (*n* = 41). Blue bars indicate the number of women who use each tool, while grey bars represent those who do not. As shown, WhatsApp is the most frequently used tool, whereas videoconferencing platforms such as Zoom, Meet, and Teams are the least utilized. (**b**). Bar chart depicting the usage frequency of digital tools among older men (*n* = 29). Blue bars correspond to the number of men who use each tool, whereas grey bars indicate those who do not. As illustrated, WhatsApp emerges as the most commonly used tool, while PowerPoint is the least frequently used.

Figure 2a,b displays the frequency of digital tool usage by gender, revealing notable differences in adoption patterns and preferences. Women show a marked tendency toward communication and social platforms, with universal use of WhatsApp (100%) and high engagement in social media (77.5%). In contrast, men report greater use of productivity-related tools, such as Excel (36.7%) and Zoom/Meet/Teams (33.3%), which are typically associated with professional or academic environments.

These findings suggest that older women are more inclined to use digital tools for social and personal communication, whereas older men engage more with work or task-oriented technologies. Although both groups show relatively low usage rates of Email and Online Banking, men report slightly higher engagement with these services.

**Figure 3:**
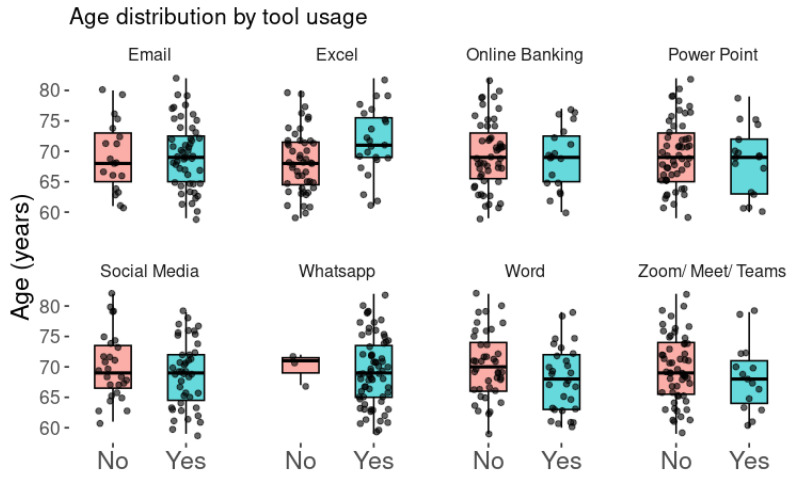
Age distribution by tool usage. The graph shows the age distribution according to the use of various digital tools by older adults. The groups of older adults who do not use each tool are shown in red, and those who do use it are shown in blue. The box plots illustrate the median, quartiles, and age distribution, with individual data points representing each participant.

Figure 3 shows digital tool usage in relation to age reveals certain behavioral patterns. Generally, younger older adults tend to make more frequent use of platforms such as Zoom, Word, and Excel, likely because these tools are commonly required in professional or academic contexts. Their familiarity with these platforms may stem from regular use in the workplace or during their education. In contrast, tools like WhatsApp and social media are used across a broader age range. While younger older adults are still the majority, it is clear that many older adults have also adopted these platforms—particularly WhatsApp, which seems to have become a common communication method for older people.

For other tools, such as PowerPoint or online banking, age differences are less pronounced. This may suggest that their usage depends more on specific needs or circumstances rather than age itself. In summary, age appears to be a relevant factor in determining which digital tools people use, especially those that require technical skills or are linked to formal settings.

### 6.2. Reliability and Validity

Table 3 presents the results of the reliability and validity assessment of the constructs. The CR values for the constructs range from 0.847 to 0.929, exceeding the recommended threshold of 0.7 [48]. Likewise, Cronbach’s alpha values ranged from 0.716 to 0.860, confirming adequate internal consistency of the constructs.

According to the criterion established by [48], multicollinearity is considered problematic when the Variance Inflation Factor (VIF) exceeds a value of 5, indicating a strong linear relationship among predictor variables. In this analysis, the VIF values were ANX1 (1.575), ANX2 (1.575), PEU1 (1.633), PEU2 (2.030), PEU3 (1.930), IS1 (2.319), IS2 (2.319), IU1 (1.451), IU2 (1.451), UP1 (1.565), UP2 (1.660), and UP3 (2.237). Since all values are well below the critical threshold, multicollinearity does not appear to be a concern in this model. Therefore, the predictor variables demonstrate sufficient statistical independence, supporting the validity of the model’s estimates.

Regarding convergent validity, the AVE coefficients ranged from 0.689 to 0.867, meeting the minimum criterion of 0.5. This suggests that the indicators explain a significant proportion of the construct’s variance. The factor loadings of the observed variables are all above 0.7, with values ranging from 0.725 (IU1) to 0.976 (IU2), further supporting convergent validity.

Based on the results presented, discriminant validity can be evaluated by applying Fornell and Larcker’s [46] criterion, which establishes that the square root of the average variance extracted (AVE) of a construct must be greater than any correlation between that construct and the others. The analysis is shown in Table 4 below.

### 6.3. Causal Model

In this study, the results obtained for the endogenous variables of the model were as follows: Perceived Usefulness had an R^2^ of 0.569, indicating that the model explains 56.9% of the variance in the user’s perception of the technology’s usefulness. This high level of explained variance reflects a strong influence of the independent variables and reinforces the robustness of the proposed model, consistent with prior studies in technology acceptance [48].

Perceived Ease of Use showed an R^2^ of 0.334, meaning that 33.4% of its variance is explained by the model. This suggests a moderate-to-high impact of the independent variables on this construct, aligning with findings from earlier TAM-based research.

Lastly, Behavioral Intention to Use exhibited an R^2^ of 0.481, indicating that the model accounts for 48.1% of the variance in users’ intention to adopt the technology. This value also reflects a substantial explanatory capacity and underscores the predictive relevance of the model’s constructs.

**Figure 4:**
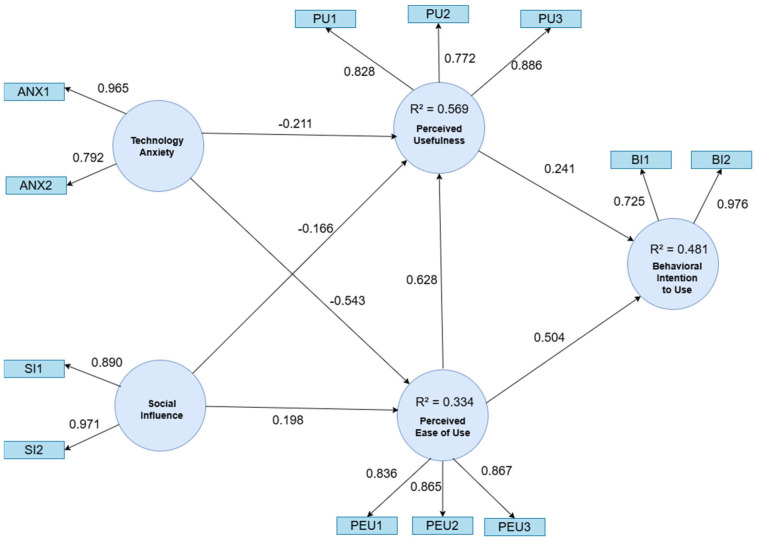
Resulting model. Anxiety (ANX), Social Influence (SI), Perceived Usefulness (PU), Perceived Ease of Use (PEOU), and Behavioral Intention to Use (BI).

Overall, these results confirm that the model demonstrates moderate to high explanatory power across the three endogenous variables. The high R^2^ values for Perceived Usefulness and Behavioral Intention to Use suggest a robust predictive performance, while the moderate value for Perceived Ease of Use is consistent with trends observed in prior empirical studies. Figure 4 graphically presents the resulting model, facilitating a clearer interpretation of the relationships between factors.

Table 5 presents the causal relationships evaluated in the structural model, specifying the standardized coefficients (β) for each relationship, along with the acceptance or rejection of the proposed hypotheses. These relationships show the interaction between the constructs within the model and provide clear evidence to understand the direction and intensity of the effects hypothesized.

Table 6 presents the effect size (f^2^) values for the structural paths. The strongest effects were observed for Perceived Ease of Use → Perceived Usefulness (f^2^ = 0.610) and Anxiety → Perceived Ease of Use (f^2^ = 0.442), both categorized as large. Medium effects were found for Perceived Ease of Use → Behavioral Intention to Use (f^2^ = 0.241), while the remaining paths showed small but relevant effects.

As shown in Table 7, the overall fit of the model was evaluated using several indices. The Standardized Root Mean Square Residual (SRMR) was found to be 0.20, which is slightly higher than the commonly accepted threshold of 0.08 [50], suggesting that the model fit could be improved. Likewise, the Normed Fit Index (NFI) yielded a value of 0.552, falling short of the recommended cut-off of 0.90. The chi-square statistic was 262.397, although this measure is generally considered of limited use in PLS-SEM due to its sensitivity to sample size and model complexity. The discrepancy measures d_ULS (1.396) and d_G (0.728) were also reported, which do not have standard cut-off points and are mainly used for comparative purposes between models.

Despite these values, it is important to note that PLS-SEM places greater emphasis on the model’s predictive power and explanatory relevance rather than on global fit indices. Therefore, the results should be interpreted considering the explained variance (R^2^), the strength and significance of path coefficients, and the effect sizes (f^2^), all of which offer more robust evidence regarding the quality of the model [48,51].

## 7. Discussion

The results of this study identify how technological anxiety and social influence affect the intention to use digital technologies among older adults in Mexico City, a context characterized by inequalities in access to and use of ICTs. It was found that technological anxiety acts as a significant barrier that reduces willingness to use technology, while the influence of close people such as family and friends does not have a positive impact on the perception of usefulness, contrary to what has been proposed in previous studies. These findings reveal the importance of considering specific cultural factors that can modify factors such as social influence. The discussion that follows examines these relationships, contrasting them with the existing literature.

Hypotheses H1 and H2 were validated, as the results indicate that anxiety represents a significant barrier to ICT adoption, causing older adults to perceive technology as less easy to use and not clearly recognize the benefits it can bring to their lives. This finding is consistent with previous studies [10,28], which identify fear of making mistakes and lack of confidence as key factors limiting older adults’ willingness to use digital tools.

In the Mexican context, technological anxiety is accentuated by a structural digital divide that disproportionately affects the older adult population. Many of them do not have access to digitally literate programs designed in an inclusive manner that take into account their learning rhythms, previous experiences, and specific needs. In addition, the digital learning process is often mediated by younger family members in informal settings and without adequate pedagogical support. This type of interaction can generate feelings of dependence, frustration, or embarrassment, especially when performed impatiently. Far from empowering, these dynamics reinforce feelings of incompetence and exclusion. Cultural factors also come into play, as older adults in Mexico are unfamiliar with technological teaching processes and, in many cases, perceive the digital environment as alien or unnecessary in their daily lives [52].

This scenario reinforces the need to design culturally sensitive strategies that address not only the development of technical skills but also the emotional and social dimensions of the relationship with technology. Creating inclusive, non-intimidating environments where mistakes are normalized can help reduce technological anxiety and promote effective digital inclusion for this social group.

Regarding the analysis of social influence, hypothesis H3, which proposed that social influence would have a positive effect on perceived usefulness among older adults, was rejected. This result showed a difference from the results of previous studies, such as those by Choudrie et al. [30] and Guner and Acarturk [10], which highlight a positive social influence on perceived usefulness.

The results indicate a negative coefficient of −0.160, suggesting that the influence of family members and other significant people does not contribute to the perception of usefulness. As noted, although social influence was initially expected to have a positive effect on the perception of the usefulness of digital tools, the results revealed a negative relationship. Unlike previous studies reporting a positive association, our findings suggest that, in the case of older adults, social pressure from others may decrease rather than improve their perception of the usefulness of a technology.

This divergence from previous research can be better understood from the perspective of the specific cultural and social context of older adults in Mexico City. In this environment, the process of technology adoption is determined not only by individual attitudes but also by intergenerational dynamics and cultural values. While family members, particularly younger ones, often act as digital facilitators, their support can create tension. When guidance is given in a hurried or corrective manner, older adults may perceive it as criticism rather than encouragement, leading them to show resistance.

Culturally, in Mexico, older people are traditionally considered figures of authority and respect within the family. Receiving instructions from younger family members can disrupt this social role, making the learning process uncomfortable or even discouraging [52,53]. In addition, the lack of inclusive digital literacy programs tailored to older users can reinforce the perception that technology is not for them, further weakening their sense of usefulness.

On the other hand, hypothesis H4 is confirmed, demonstrating that the opinions of the social environment, such as family and friends, significantly influence the perception that technology is easy to use. This finding is consistent with international studies, such as that by Guner and Acarturk [10] in Türkiye, who pointed out that social support favors the perception of ease of use. Similarly, Choudrie et al. [30] and Hoque and Sorwar [31] have documented that the influence of family members and close friends is a critical factor in the technological adoption of older adults. In the Mexican case, individual decisions, especially among older adults, are deeply influenced by the family, which is why the role of the family nucleus as a promoter of technology use can be decisive in contexts where digital literacy is limited.

Hypotheses H5 and H7 were also confirmed, indicating that both perceived usefulness and perceived ease of use have a positive effect on intention to use. This finding reinforces the original TAM postulates proposed by Davis [8], as well as more recent research in different cultural contexts, such as that of Martín García et al. [9] in Spain and Yau and Hsiao [11] in Taiwan. The consistency of results suggests that, regardless of country or culture, older adults tend to adopt technology when they consider it useful for improving their daily activities and when it does not represent a significant cognitive burden. However, in Mexico, where digital divides are greater due to factors such as low access to technological training and a lack of adequate infrastructure, this perception of usefulness and ease of use may be mediated by previous negative experiences or by dependence on third parties to solve technical problems.

This indicates that technological inclusion strategies must be accompanied by interventions that reduce uncertainty and strengthen the autonomy of older adults.

Hypothesis H6 was also confirmed, indicating that the perception of ease of use has a positive effect on the perception of usefulness. The coefficient obtained (β = 0.691) reflects a strong relationship, meaning that when older adults consider technology to be easy to use, they also perceive it as useful. This result is in line with studies such as those by Zin et al. [34], Yau and Hsiao [11], and Aguilar-Flores et al. [35], which highlight how reducing technical complexity improves the functional assessment of technological tools. In a context such as Mexico, where many older adults do not have early contact with technology, this relationship may be accentuated. In other words, the perception of usefulness could depend almost entirely on the experience of use, in contrast to countries with more established digital literacy trajectories.

This suggests that facilitating the first approach through accessible devices, user-friendly environments, or support programs improves not only the user experience but also its functional assessment.

Taken together, these findings confirm the validity of the Technology Acceptance Model in the Mexican case but also highlight the importance of incorporating contextual variables into the design of digital inclusion strategies for older adults. The role of family, perceived ease, and usefulness cannot be analyzed in isolation, as they are connected to the social environment of Mexico City.

These cultural dynamics may help explain why social influence in this study negatively affected perceived usefulness. Rather than motivating adoption, peer pressure can trigger defensive attitudes or diminish self-efficacy. To counteract this, technology inclusion strategies should emphasize empathetic learning between generations and respect the cultural identities and social roles of older adults.

### 7.1. Theoretical Contribution

This study makes a significant theoretical contribution by deepening the understanding of the TAM in a culturally specific and underexplored of population older adults in Mexico City. While TAM has been widely validated across diverse contexts, its application among older adults in Latin America remains limited. The finding that technological anxiety acts as a substantial barrier to both perceived usefulness and perceived ease of use refines TAM by emphasizing the emotional dimensions of technology interaction, particularly among older users who have limited prior exposure to digital environments. Moreover, the dual role of social influence, which enhances perceived ease of use but diminishes perceived usefulness, challenges conventional assumptions embedded in TAM and related models such as the Unified Theory of Acceptance and Use of Technology (UTAUT). Traditional approaches tend to posit social influence as a uniformly positive factor in technology adoption. However, this study demonstrates that in contexts marked by strong generational hierarchies and cultural norms such as those found in Mexican families, social pressure can act as a disincentive. This offers a more complex, culturally embedded perspective on social influence and underscores the importance of contextualizing theoretical models to better reflect the lived experiences of older adults. In this way, the research reinforces the need to adapt the TAM to local contexts by incorporating sociocultural dynamics, intergenerational relationships, and emotional factors as core components of the model. These contributions provide a basis for developing more inclusive theoretical models that reflect the diversity of aging populations and their interactions with digital technologies across different cultural and socioeconomic contexts.

### 7.2. Practical Contribution

From a practical standpoint, the results of this study are relevant for policy design, digital inclusion programs, educational strategies, and technological development aimed at older adults in Latin America and similar contexts.

First, the strong negative impact of technological anxiety suggests that digital inclusion strategies should go beyond technical training and prioritize emotional resilience, self-efficacy, and digital safety. Programs should be designed to foster positive emotional experiences through supportive learning environments, patient instruction, and normalization of mistakes. This also implies rethinking training environments to ensure that older adults feel competent, respected, and autonomous.

Second, the ambiguous role of social influence, which favors ease of use but undermines perceived usefulness, has important practical implications. Digital literacy initiatives should be designed to empower, rather than pressure, older adults. For example, intergenerational programs should be structured to encourage empathetic and collaborative learning rather than hierarchical and corrective instruction. This may require training younger family members or facilitators in pedagogical approaches that take into account the needs and characteristics of this population.

Interfaces should be simplified, visual and textual elements should be culturally and linguistically adapted, and usability should be tested with older adults from diverse socioeconomic backgrounds. In addition, the availability of technical support and infrastructure should be improved to maintain ongoing commitment to digital inclusion.

Finally, the results can serve as a basis for the formulation of public policies for digital inclusion. Governments and institutions should fund and promote initiatives that help older adults stay connected in the digital society and acquire digital skills. This includes both access to devices and connectivity, as well as the creation of lifelong learning ecosystems.

## 8. Conclusions

This study addressed the following research question: How do technological anxiety and social influence affect older adults’ willingness to use technology in Mexico City? The results show that technological anxiety has a significant negative impact, as it reduces both the perceived ease of use and perceived usefulness of digital technologies, which in turn decreases the intention to adopt them. In contrast, social influence demonstrated a dual effect: while it positively influences perceived ease of use, it negatively affects perceived usefulness, contradicting previous results obtained in other contexts and revealing that older adults’ willingness to use technology in Mexico City is strongly conditioned by specific emotional barriers and sociocultural dynamics.

Cultural and societal factors in Mexico play an important role in shaping older adults’ technology anxiety and the social influence they experience. Socioeconomic inequalities, marked by poverty and limited digital infrastructure, heighten anxiety by making access to ICTs costly and unpredictable [2,54]. Geographic disparities, especially in rural areas, further exacerbate this concern, as high connectivity costs and few service options fuel frustration over potential technical failures [55].

As we can see, the digital divide among older adults in Mexico represents a significant challenge, as it limits their participation in an increasingly digital society and restricts their access to essential services such as healthcare, government services, and online banking. Despite technological advances, many older adults remain disconnected due to barriers such as limited digital skills, unequal access to education, and, especially, technology anxiety, which remains a major deterrent to their participation in digital tools.

One of the most important findings is the role of intergenerational family ties, which have a dual effect. Younger relatives can reduce anxiety by providing guidance and practical support, but when that assistance is rushed or impatient, it can increase technology anxiety and undermine perceived usefulness, discouraging continued use. Therefore, in a context where the family is the primary source of social support, it is essential to design interventions that empower older adults rather than pressure them.

To effectively address this issue, it is important to develop strategies that reduce technological anxiety and promote a sense of security and self-efficacy. Gradual, adaptive training that respects individual learning rhythms, coupled with simple, empathetic communication, helps build confidence and reduces fear of failure. Educational materials used for digital literacy should avoid cognitive overload and focus on building confidence through repetition, clarity, and practice [1].

In an increasingly digital world, enabling older people to participate fully is not just a technological or educational issue but a social and ethical imperative, as promoting digital inclusion improves autonomy and quality of life and reduces social isolation. To achieve this, an approach is needed that integrates thoughtful pedagogical design, culturally sensitive social support, and the development of accessible technologies. Intergenerational collaboration, in which young people guide older people in digital literacy, strengthens social ties and encourages informal learning [56], and when combined with the use of clear and accessible language that avoids technical terms, it can further facilitate understanding, minimizing frustration among this population.

Finally, it is important to note that only through collective action by governments, educational institutions, technology developers, and, especially, families can we reduce the digital divide and help ensure that everyone, regardless of age, can benefit from the opportunities offered by the digital age.

### Limitations and Directions for Future Research

This study presents limitations that should be considered when interpreting the findings. First, the sample size was relatively small (70 older adults) and drawn from a single event held in Mexico City, which limits the generalizability of the results to other regions or demographic contexts within the country. Additionally, the quantitative approach used in this research does not allow for an in-depth exploration of individual experiences or the more complex sociocultural barriers that may influence technology adoption among older adults.

Future research should aim to expand the sample size and include participants from diverse geographical regions and socioeconomic backgrounds across Mexico. It would also be valuable to employ a mixed-methods or qualitative approach to gain deeper insights into older adults’ attitudes, emotions, and motivations regarding digital technology use. Future studies could assess the effectiveness of targeted interventions aimed at reducing technology anxiety and enhancing supportive social environments, with the goal of promoting more inclusive and sustainable digital integration for the aging population.

## Figures and Tables

**Table 2:** Items used in the technology use survey in older adults.

Construct	Code	Items	Reference
Anxiety	ANX1	I feel apprehensive about using ICT	Venkatesh et al. [14]
	ANX2	It scares me to think that I could lose a lot of information using ICT by hitting the wrong key.	
Social Influence	IS1	People who are important to me think that I should use ICT	Venkatesh et al. [14]
	IS2	People whose opinions are valuable to me prefer that I use ICT	
Perceived Usefulness	PU1	Using ICT in my job would increase my productivity.	Davis [8] Venkatesh & Davis [43] Venkatesh et al. [14]
	PU2	Using ICT would improve my job performance.	
	PU3	I would find ICT useful in my activities.	
Perceived Ease of Use	PEU1	Learning to operate ICT would be easy for me.	Davis [8] Venkatesh & Davis [43] Venkatesh et al. [14]
	PEU 2	I would find it easy to get ICT to do what i want it to do.	
	PEU 3	I find ICT easy to use.	
Behavioral intention to use	IU1	Assuming I have access to ICT, I intend to use it.	Davis [8] Venkatesh & Davis [43] Venkatesh & Bala [44]
	IU2	Given that I have access to ICT, I predict that I would use it.	

**Table 3:** Construct validity through composite reliability and the mean of the variance extracted.

Latent Factor	Cronbach’s alpha (A)	Composite Reliability (CR)	Average Variance Extracted (AVE)	Observed Variable	Factor Loading	Media	Standard Deviation
Anxiety	0.753	0.875	0.779	ANX1	0.965	0.966	0.013
				ANX2	0.792	0.787	0.071
Social Influence	0.860	0.929	0.867	IS1	0.890	0.881	0.104
				IS2	0.971	0.956	0.066
Perceived Usefulness	0.775	0.869	0.689	PU1	0.828	0.827	0.040
				PU2	0.772	0.778	0.054
				PU3	0.886	0.883	0.038
Perceived Ease of Use	0.818	0.892	0.733	PEU1	0.836	0.839	0.032
				PEU 2	0.865	0.865	0.034
				PEU 3	0.867	0.865	0.025
Behavioral intention to use	0.716	0.847	0.739	IU1	0.725	0.701	0.143
				IU2	0.976	0.976	0.011

**Table 4:** Discriminant validity analysis.

	Anxiety	Social Influence	Perceived Usefulness Utilidad	Perceived Ease of Use Facilidad	Behavioral Intention to Use
Anxiety	**0.883**				
Social Influence	−0.002	**0.931**			
Perceived Usefulness	−0.552	−0.040	**0.830**		
Perceived Ease of Use	−0.543	0.199	0.710	**0.856**	
Behavioral intention to use	−0.432	0.248	0.597	0.673	**0.860**

**Table 5:** Summary of significant relationships in the structural model.

Causal Relationship (Path)	Coef β	Hypothesis
ANX → PU	β = −0.158	H1 = Accepted
ANX → PEOU	β = −0.567	H2 = Accepted
SI → PU	β = −0.160	H3 = Rejected
SI → PEOU	β = 0.249	H4 = Accepted
PU → BI	β = 0.240	H5 = Accepted
PEOU → PU	β = 0.655	H6 = Accepted
PEOU → BI	β = 0.504	H7 = Accepted

**Table 6:** Effect size (f^2^) of the structural model.

Causal Relationship (Path)	f^2^ Value	Effect Size
ANX → PU	0.072	Small
ANX → PEOU	0.442	Large
SI → PU	0.060	Small
SI → PEOU	0.059	Small
PU → BI	0.055	Small
PEOU → PU	0.610	Large
PEOU → BI	0.241	Medium

**Table 7:** Summary of model fit.

Fit Index	Value
SRMR (Standardized Root Mean Square Residual)	0.20
NFI (Normed Fit Index)	0.552
Chi-square (χ^2^)	262.397
d_ULS	1.396
d_G	0.728

## Data Availability

The original contributions presented in this study are included in the article. Further inquiries can be directed to the corresponding author.

## Abbreviations

The following abbreviations are used in this manuscript:

TAM	Technology Acceptance Model.
ICT	Information and Communications Technologies.
